# Inflammatory myofibroblastic tumour of the lung: Case report and review of the literature

**DOI:** 10.1002/rcr2.885

**Published:** 2021-12-07

**Authors:** Sonila Boriçi, Marjeta Tanka, Luljeta Serbo

**Affiliations:** ^1^ Service of Pulmonology and Allergy University Hospital Center “Mother Tereza” Tirana Albania

**Keywords:** follow‐up, inflammatory, lung, myofibroblastic, tumour

## Abstract

Inflammatory myofibroblastic tumour (IMT) of the lung is a rare tumour encountered in children. Although it is seen mostly in paediatric ages, a small number of cases exists in the literature. It may appear as an inflammatory mass or may have the characteristics of a tumour with the ability for recidivism and metastasis. Careful follow‐up of the cases is required to differentiate between the two. We present the case of a 4‐year‐old girl who presented with cough, and the chest x‐ray and computed tomography scan revealed a tumour mass in the right lung. After lobectomy, histological examination combined with immunohistochemical study discovered an IMT of the lung.

## Graphical abstract

Inflammatory myofibroblastic tumour of the lung is a rarely encountered lesion. Although it is commonly seen in children, the number of childhood cases in the literature is limited. It has a dual nature: it may behave as an inflammatory lesion or a tumour with the capacity for recidivism and metastasis. So, the syndrome requires careful follow‐up.

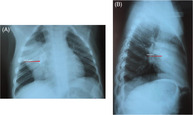

## INTRODUCTION

Inflammatory myofibroblastic tumour (IMT) of the lung is a rare finding, comprising less than 1% of all surgically resected lung lesions. Nevertheless, in paediatric ages, it is a common primary lung tumour and is usually found incidentally.^1^, ^2^, ^3^, ^4^, ^5^, ^6^, ^7^

IMT of the lung can be presented as an isolated mass but it can also be locally invasive.^1^, ^8^, ^9^, ^10^ It is still a dilemma for these tumours, whether they represent a pure inflammatory process or a low‐grade malignancy with a dominant inflammatory response. Here, we describe the management of a case and also a review of the literature.

## CASE REPORT

**FIGURE 1:**
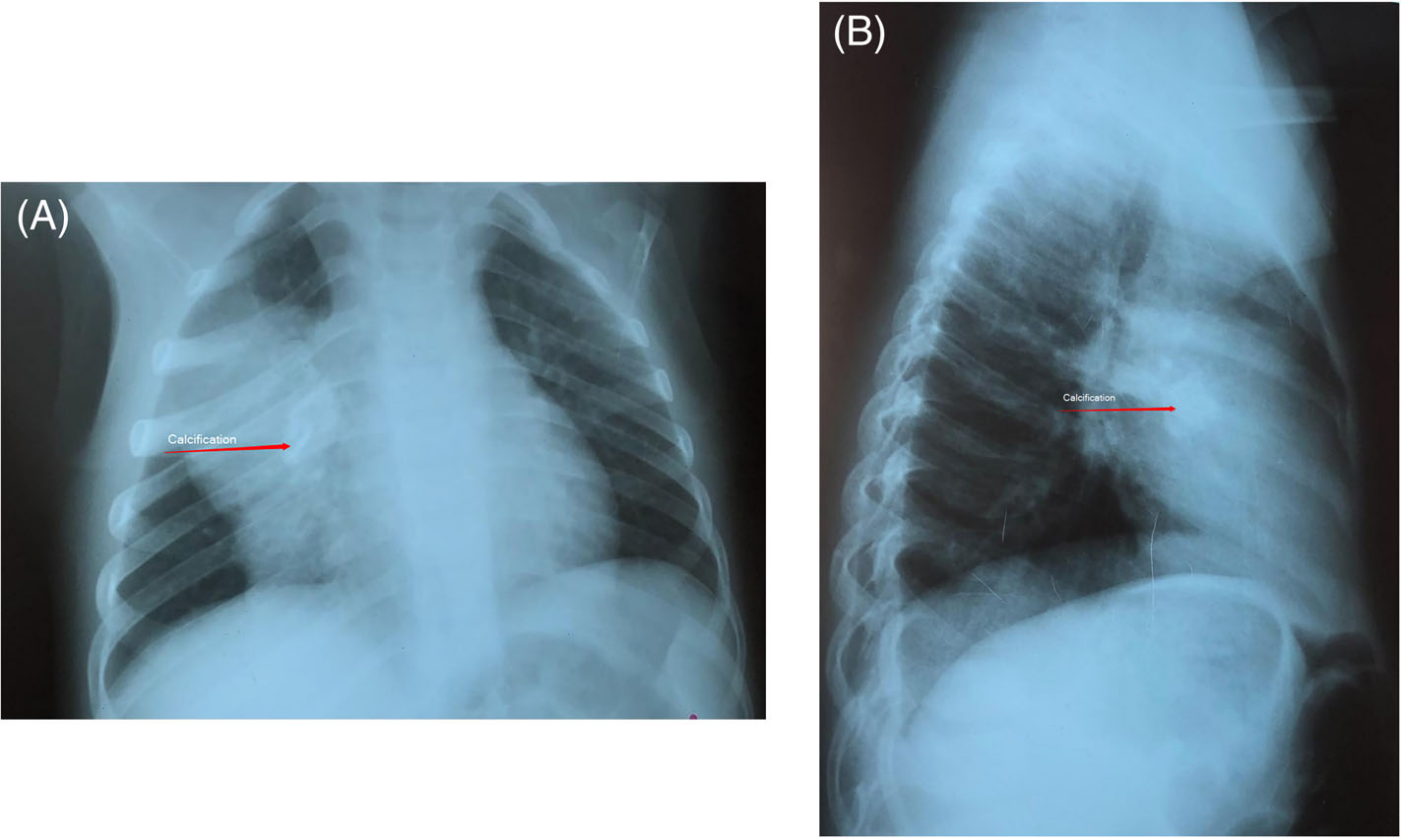
(A) Thorax radiography of the myofibroblastic tumour. (B) Lateral radiography of thorax shows myofibroblastic tumour of the lung

**FIGURE 2:**
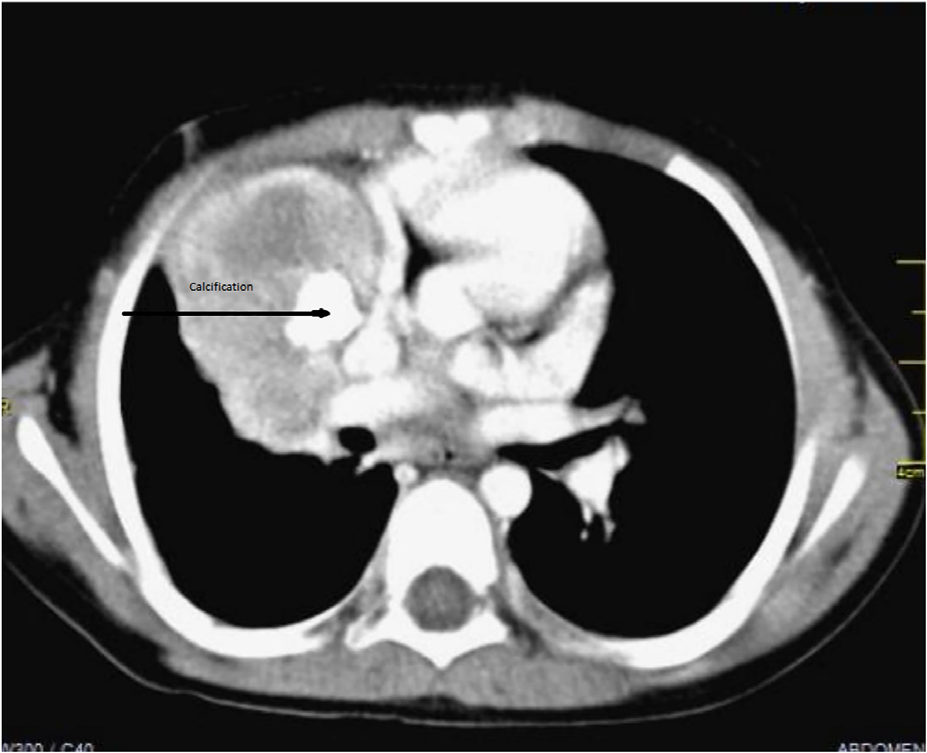
Computed tomography of the myofibroblastic tumour of the lung

A 4‐year‐old girl was referred to our tertiary hospital for the specialist evaluation of a non‐specific cough, which was present for 6 weeks, associated with a right pulmonary mass. The physical examination was normal; on auscultation, diminished breath sounds were present on the right side of the lung. The medical history and growth were unremarkable. Chest radiograph revealed a mass located in the right upper lobe and the middle lobe of the right lung, with a central area of calcification (Figure 1). Chest computed tomography (CT) scan confirmed the chest radiograph findings; a solid, well‐contoured, heterogeneous, mass was noted in the right upper lobe and middle lobe of the lung with an area of central calcification (Figure 2).

No lymphadenopathy was detected. Microscopy, culture and cytology of the sputum were unremarkable.

The erythrocyte sedimentation rate was 10, haemoglobin was 11.6 g/dl and the leucocyte count was 8.8 *×* 10^9^/L. The other serum haematological and biochemical results were normal. The serology of Echinococcus and Mantoux test were negative.

The patient did not respond to antibiotics; therefore, surgical removal of the mass was performed. Thoracotomy performed on the right side showed a lesion in the lung parenchyma, extended to the upper and middle lobes. The differential diagnosis of congenital lesions of the lung was made. As there was no success with fine‐needle aspiration in several cases, we decided to perform surgical resection.

The lesion was resected and lobectomy of both upper lobe and middle lobe was also performed. No associated lymphadenopathy was noted.

Macroscopically, a well‐circumscribed mass measuring 5.5 *×* 5.5 *×* 4 cm was present. The excised tumour had an osseous centre measuring 2 *×* 1 *×* 1 cm. From the histological point of view, the mass consisted of disorganization of the normal bronchoalveolar parenchyma, myofibroblastic cells and inflammatory cell infiltrates, such as lymphocytes, neutrophils, eosinophils and histiocytes. In the centre of the lesion, a large ossification was found.

Immunohistochemistry revealed calponin antigens, SMA, desmin, P53, bcL2, CK, HMW, and, in several myofibroblastic cells, expression of ALK‐1. It was negative for beta‐catenin, cyclin D1, Myo‐D1, S100p, CD34, CD99 and CD117.

The histological and immunohistochemistry characteristics were compatible with an inflammatory myofibroblastic pseudotumor.

The post‐operative course was uneventful, and the patient was discharged home after 2 weeks of surgery. The patient re‐presented to the hospital 1 year after surgery. The child had normal daily activities, normal chest x‐ray and no recurrences.

## DISCUSSION

IMT is a rare tumour of the lungs, which was first described in 1939.^8^ They can grow to the lung, the brain, skin, soft tissues, liver and so on.^8^, ^9^, ^10^, ^11^, ^12^, ^13^, ^14^, ^15^ They develop mostly in paediatric ages.^2^, ^3^, ^4^, ^5^, ^6^, ^7^ The aetiology and pathogenesis are not fully understood. Some hypotheses support an abnormal immunological response towards viruses or foreign antigens; herpes type 8 virus and Epstein–Barr are mostly accused.^16^, ^17^, ^18^ Immunohistochemical results of IMT show the presence of polyclonal plasma cells, which support the fact that IMT is an inflammatory process.^17^, ^18^ Based on histopathological data, the other hypothesis presents IMT as low‐grade neoplasms with a superimposed inflammation.^19^

The myofibroblast is recognized as the principal cell type of IMTs.^20^ Because of variable histology results, several synonyms are used for these masses, such as: plasma cell granuloma, fibrous histiocytoma, inflammatory pseudo‐tumour, fibroxanthoma and xanthogranuloma.

IMTs are usually benign tumours, mainly occurring in paediatric ages. They are one of the most common lung tumours in patients younger than 16 years.^1^, ^2^, ^3^, ^4^, ^5^, ^6^, ^7^ Recurrence and/or malignant transformation of myofibroblastic tumour is a rare phenomenon. Markers for the neoplasia development include chromosome 2p23 reordering and ALK‐1 expression.^21^ Coffin et al.^17^ showed that ALK‐1 may be a marker of recidivism, but not a marker of malignant transformation. In the study by Chun et al.,^18^ the authors found a better prognosis in patients who tested positive for ALK‐1. Meanwhile, recurrences may be attributed to incomplete resection of the mass.

These lesions have no gender difference, and represent less than 1% of lung tumours.^1^, ^2^, ^3^, ^4^, ^5^, ^6^, ^7^ Most of the cases have no symptoms and are discovered incidentally by chest radiograph.^17^

Physical examination and laboratory tests demonstrated no specific findings. The chest radiograph usually demonstrates a mass of 1–10 cm in diameter.^23^ Multiple nodules, calcifications and invasive disease have been reported occasionally.^24^ The diagnosis cannot be relied on chest CT and/or bronchoscopy and fine‐needle aspiration biopsy. Surgery is recommended for diagnostic and therapeutic reasons.^25^, ^26^

Macroscopically, IMTs are firm yellow‐white masses, have clear borders and may also have calcifications. They are usually located in the parenchyma, and sometimes may be located as endobronchial masses.

Microscopically, the lesions have fibroblasts, myofibroblasts, lymphocytes, plasma cells, histiocytes, neutrophils and eosinophils.^20^ Immunohistochemistry demonstrates the predominance of immunoglobulin G.^17^, ^18^

The diagnosis should be based on careful histological examination and even immunohistochemistry stains several times.^20^, ^27^ If possible, resection of the whole mass is recommended. To guide the extent of excision, frozen sections obtained at the time of operation time can be helpful, generally minimized to preserve lung function.^28^

The differential diagnosis includes pseudolymphoma, lymphosarcoma and fibrous histiocytoma.^18^, ^20^, ^27^, ^29^

Surgical resection of the whole mass is recommended, in order to exclude malignancy and completely treat the disease.^18^, ^24^, ^26^, ^30^ In cases when complete surgical resection is not possible, in multifocal disease or if recurrences occur, treatment options are radiotherapy, corticosteroids, chemotherapy and competitive inhibitors of ALK tyrosine kinase.^3^, ^18^, ^31^

The prognosis of myofibroblastic tumour is very good after total surgical removal, and the chances for recurrence and malignant transformation are low. Long‐term follow‐up is recommended after surgery.^23^

Clinicians need to be aware about the different clinical presentations of lung IMTs, which are rare, usually asymptomatic and may present as a pulmonary mass with well‐defined borders that may resemble cancer. The best method to diagnose and treat them is surgical excision. As preoperative tests are not diagnostic, tumour's excision is necessary to exclude malignancy. When possible, total excision of the mass is recommended in order to prevent recurrence of the tumour.

## CONFLICT OF INTEREST

None declared.

## AUTHOR CONTRIBUTION

Sonila Boriçi: conception or design of the work; acquisition, analysis or interpretation of the data; drafting the work or revising it critically for important intellectual content; and final approval of the version to be published. Marjeta Tanka: drafting the work or revising it critically for important intellectual content. Luljeta Serbo: drafting the work or revising it critically for important intellectual content.

## ETHICS STATEMENT

The authors declare that appropriate written informed consent was obtained for the publication of this manuscript and accompanying images.

## Data Availability

The data that support the findings of this study are available from the corresponding author upon reasonable request.

## References

[1] Ishida T , Oka T , Nishino T , Tateishi M , Mitsudomi T , Sugimachi K . Inflammatory pseudotumor of the lung in adults: radiographic and clinicopathological analysis. Ann Thorac Surg. 1989;48:90–5. 2764605 10.1016/0003-4975(89)90187-2

[2] Copin MC , Gosselin BH , Ribet ME . Plasma cell granuloma of the lung: difficulties in diagnosis and prognosis. Ann Thorac Surg. 1996;61(5):1477–82. 8633962 10.1016/0003-4975(96)00081-1

[3] Koea JB , Broadhurst GW , Rodgers MS , McCall JL . Inflammatory pseudotumor of the liver: demographics, diagnosis, and the case for nonoperative management. J Am Coll Surg. 2003;196:226–35. 12595051 10.1016/S1072-7515(02)01495-3

[4] Aizawa T , Sato T , Tanaka Y , Kishimoto K , Watanabe M , Kokubun S . Intramedullary plasma cell granuloma in the cervicothoracic spine. Case report. J Neurosurg. 2002;97:235–8. 12296686 10.3171/spi.2002.97.2.0235

[5] Lewis JT , Gaffney RL , Casey MB , Farrell MA , Morice WG , Macon WR . Inflammatory pseudotumor of the spleen associated with a clonal Epstein‐Barr virus genome. Case report and review of the literature. Am J Clin Pathol. 2003;120:56–61. 12866373 10.1309/BUWN-MG5R-V4D0-9YYH

[6] Lee SH , Fang YC , Luo JP , Kuo HI , Chen HC . Inflammatory pseudotumour associated with chronic persistent *Eikenella corrodens* infection: a case report and brief review. J Clin Pathol. 2003;56:868–70. 14600136 10.1136/jcp.56.11.868 PMC1770116

[7] Ceruse P , Ramade A , Vautrin R , Crozes C , Dubreuil C , Disant F . Inflammatory pseudotumor of the neck: a long‐term result without surgical approach. Otolaryngol Head Neck Surg. 2005;132:812–3. 15886643 10.1016/j.otohns.2004.09.054

[8] Nonomura A , Mizukami Y , Matsubara F , Shimizu J , Oda M , Watanabe Y , et al. Seven patients with plasma cell granuloma (inflammatory pseudotumor) of the lung, including two with intrabronchial growth: an immunohistochemical and electron microscopic study. Intern Med. 1992;31:756–65. 1392177 10.2169/internalmedicine.31.756

[9] Vancauwenbergh A , Smet MH , Breysem L . Inflammatory pseudotumor of the lung. JBR‐BTR. 2002;85:209–11. 12403391

[10] Urschel JD , Horan TA , Unruh HW . Plasma cell granuloma of the lung. J Thorac Cardiovasc Surg. 1992;104:870–5. 1405683

[11] Lebecque P , Lapierre JG , Brochu P , Spier S , Lamarre A . Pulmonary plasma cell granuloma. Eur J Pediatr. 1987;146:174–6. 3569355 10.1007/BF02343227

[12] Pettinato G , Manivel JC , De Rosa N , Dehner LP . Inflammatory myofibroblastic tumor (plasma cell granuloma). Clinicopathologic study of 20 cases with immunohistochemical and ultrastructural observations. Am J Clin Pathol. 1990;94:538–46. 2239820 10.1093/ajcp/94.5.538

[13] Souid AK , Ziemba MC , Dubansky AS , Mazur M , Oliphant M , Thomas FD , et al. Inflammatory myofibroblastic tumor in children. Cancer. 1993;72:2042–8. 8364883 10.1002/1097-0142(19930915)72:6<2042::aid-cncr2820720641>3.0.co;2-i

[14] Mahr MA , Salomao DR , Garrity JA . Inflammatory orbital pseudotumor with extension beyond the orbit. Am J Ophthalmol. 2004;138:396–400. 15364221 10.1016/j.ajo.2004.04.026

[15] Buccoliero AM , Caldarella A , Santucci M , Ammannati F , Mennonna P , Taddei A , et al. Plasma cell granuloma – an enigmatic lesion: description of an extensive intracranial case and review of the literature. Arch Pathol Lab Med. 2003;127:e220–3. 12683907 10.5858/2003-127-e220-PCGEL

[16] Travis WD , Brambilla E , Muller‐Hermelink HK , Harris CC , editors. World Health Organization classification of tumors. Pathology and genetics of the lung, pleura, thymus, and heart. Lyon: IARC Press; 2004.

[17] Coffin CM , Hornick JL , Fletcher CD . Inflammatory myofibroblastic tumor: comparison of clinicopathologic, histologic, and immunohistochemical features including ALK expression in atypical and aggressive cases. Am J Surg Pathol. 2007;31(4):509–20. 17414097 10.1097/01.pas.0000213393.57322.c7

[18] Chun YS , Wang L , Nascimento AG , Moir CR , Rodeberg DA . Pediatric inflammatory myofibroblastic tumor: anaplastic lymphoma kinase (ALK) expression and prognosis. Pediatr Blood Cancer. 2005;45(6):796–801. 15602716 10.1002/pbc.20294

[19] Bahadori M , Liebow AA . Plasma cell granulomas of the lung. Cancer. 1973;31(1):191–208. 4683037 10.1002/1097-0142(197301)31:1<191::aid-cncr2820310127>3.0.co;2-d

[20] Gal AA , Koss MN , McCarthy WF , Hochholzer L . Prognostic factors in pulmonary fibrohistiocytic lesions. Cancer. 1994;73:1817–24. 8137205 10.1002/1097-0142(19940401)73:7<1817::aid-cncr2820730708>3.0.co;2-k

[21] Huellner MW , Schwizer B , Burger I , Fengels I , Schläpfer R , Bussmann C , et al. Inflammatory pseudotumor of the lung with high FDG uptake. Clin Nucl Med. 2010;35(9):722–3. 20706053 10.1097/RLU.0b013e3181ea33d0

[22] Weinberg PB , Bromberg PA , Askin FB . "Recurrence" of a plasma cell granuloma 11 years after initial resection. South Med J. 1987;80:519–21. 3563587 10.1097/00007611-198704000-00028

[23] Cerfolio RJ , Allen MS , Nascimento AG , Deschamps C , Trastek VF , Miller DL , et al. Inflammatory pseudotumors of the lung. Ann Thorac Surg. 1999;67(4):933–6. 10320231 10.1016/s0003-4975(99)00155-1

[24] Mandelbaum I , Brashear RE , Hull MT . Surgical treatment and course of pulmonary pseudotumor (plasma cell granuloma). J Thorac Cardiovasc Surg. 1981;82(1):77–82. 7242136

[25] Melloni G , Carretta A , Ciriaco P , Arrigoni G , Fieschi S , Rizzo N , et al. Inflammatory pseudotumor of the lung in adults. Ann Thorac Surg. 2005;79:426–32. 15680808 10.1016/j.athoracsur.2004.07.077

[26] Sakurai H , Hasegawa T , Watanabe S , Suzuki K , Asamura H , Tsuchiya R . Inflammatory myofibroblastic tumor of the lung. Eur J Cardiothorac Surg. 2004;25:155–9. 14747105 10.1016/s1010-7940(03)00678-x

[27] Dahabreh J , Zisis C , Arnogiannaki N , Katis K . Inflammatory pseudotumor: a controversial entity. Eur J Cardiothorac Surg. 1999;16:670–3. 10647841 10.1016/s1010-7940(99)00321-8

[28] Bando T , Fujimura M , Noda Y , Hirose JI , Ohta G , Matsuda T . Pulmonary plasma cell granuloma improves with corticosteroid therapy. Chest. 1994;105:1574–5. 8181356 10.1378/chest.105.5.1574

[29] Narla LD , Newman B , Spottswood SS , Narla S , Kolli R . Inflammatory pseudotumor. Radiographics. 2003;23:719–29. 12740472 10.1148/rg.233025073

[30] Fornell‐Pérez R , Santana‐Montesdeoca JM , García‐Villar C , Camacho‐García MC . Two types of presentation of pulmonary inflammatory pseudotumors. Arch Bronconeumol. 2012;48:296–9. 22075403 10.1016/j.arbres.2011.09.001

[31] Christensen JG , Zou HY , Arango ME , Li Q , Lee JH , McDonnell SR , et al. Cytoreductive antitumor activity of PF‐2341066, a novel inhibitor of anaplastic lymphoma kinase and c‐Met, in experimental models of anaplastic large‐cell lymphoma. Mol Cancer Ther. 2007;6:3314–22. 18089725 10.1158/1535-7163.MCT-07-0365

